# The Role of Metallothionein in Oxidative Stress

**DOI:** 10.3390/ijms14036044

**Published:** 2013-03-15

**Authors:** Branislav Ruttkay-Nedecky, Lukas Nejdl, Jaromir Gumulec, Ondrej Zitka, Michal Masarik, Tomas Eckschlager, Marie Stiborova, Vojtech Adam, Rene Kizek

**Affiliations:** 1 Central European Institute of Technology, Brno University of Technology, Technicka 3058/10, CZ-616 00 Brno, Czech Republic; E-Mails: brano.ruttkay@seznam.cz (B.R.-N.); lukasnejdl@gmail.com (L.N.); j.gumulec@gmail.com (J.G.); zitkao@seznam.cz (O.Z.); masarik@med.muni.cz (M.M.); vojtech.adam@mendelu.cz (V.A.); 2 Department of Chemistry and Biochemistry, Faculty of Agronomy, Mendel University in Brno, Zemedelska 1, CZ-613 00 Brno, Czech Republic; 3 Department of Pathological Physiology, Faculty of Medicine, Masaryk University, Kamenice 5, CZ-612 00 Brno, Czech Republic; 4 Department of Paediatric Haematology and Oncology, 2nd Faculty of Medicine, Charles University and University Hospital Motol, V Uvalu 84, CZ-150 06 Prague 5, Czech Republic; E-Mail: tomas.eckschlager@fnmotol.cz; 5 Department of Biochemistry, Faculty of Science, Charles University, Albertov 2030, CZ-128 40 Prague 2, Czech Republic; E-Mail: stiborov@natur.cuni.cz

**Keywords:** metallothionein, free radicals, cellular oxidative stress, zinc, transcription factor, cancer

## Abstract

Free radicals are chemical particles containing one or more unpaired electrons, which may be part of the molecule. They cause the molecule to become highly reactive. The free radicals are also known to play a dual role in biological systems, as they can be either beneficial or harmful for living systems. It is clear that there are numerous mechanisms participating on the protection of a cell against free radicals. In this review, our attention is paid to metallothioneins (MTs) as small, cysteine-rich and heavy metal-binding proteins, which participate in an array of protective stress responses. The mechanism of the reaction of metallothioneins with oxidants and electrophilic compounds is discussed. Numerous reports indicate that MT protects cells from exposure to oxidants and electrophiles, which react readily with sulfhydryl groups. Moreover, MT plays a key role in regulation of zinc levels and distribution in the intracellular space. The connections between zinc, MT and cancer are highlighted.

## 1. Introduction

Free radicals are chemical particles containing one or more unpaired electrons, which may be part of the molecule. They cause the molecule to become highly reactive [1]. Free radicals (a) can be generated during UV irradiation, X-ray or gamma radiation, (b) are the products of reactions catalyzed by metals, (c) are present in the air as pollutants, (d) are produced by neutrophils and macrophages during inflammation and (e) are by-products of the mitochondrial respiratory chain [2]. Free radicals are known to play a dual role in biological systems, because they can be considered as beneficial or deleterious [3]. The beneficial effects of free radicals are in the immune response to infection and that they are a part of many cellular signaling systems. In contrast, at high concentrations of free radicals, they may be important mediators of damage to cell structures, including lipids and membranes, proteins and nucleic acids, when oxidative stress occurs [4].

The harmful effects of free radicals are balanced by the antioxidant action of antioxidant enzymes and non-enzymatic antioxidants [5]. Despite the presence of the antioxidant defense system, which protects cells from oxidative damage originating from free radicals, oxidative damage accumulates during the lifecycle and, with radicals related damage of DNA, proteins and lipids, plays a key role in the development of diseases, such as cancer, atherosclerosis, arthritis and neurodegenerative diseases [6–8]. The most important free radicals in aerobic organisms are oxygen reactive species (ROS) [3] and nitrogen reactive species (RNS) [9]. An overview of the most important reactive oxygen and nitrogen reactive species is summarized in (Table 1). In this review, our attention is aimed at the mutual relations of metallothionein and zinc(II) ions.

## 2. Metallothioneins

**Figure 1:**
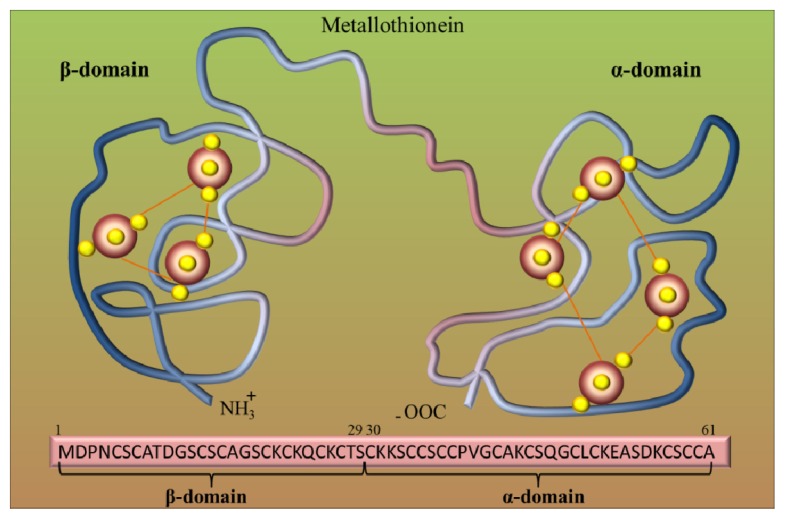
Metallothionein (MT) structure. Model of two binding sites of metallothionein. Red big beads are metal atoms (e.g., Zn), and small yellow beads are sulfur atoms. Adopted and modified according to [27].

Metallothioneins (MTs) belong to the group of intracellular cysteine-rich, metal-binding proteins that have been found in bacteria, plants, invertebrates and vertebrates [12–14]. These proteins were discovered in 1957 as cadmium-binding proteins isolated from horse kidney [15]. Since their discovery, these low molecular weight cysteine-rich proteins have been continuously studied in all aspects, including physical, chemical and biochemical properties. Mammalian MTs may contain 61–68 amino acids, and among them 20 are cysteines [16,17]. These unique proteins are involved in diverse intracellular functions [18], but their role in the detoxification of heavy metals and in the maintaining of essential metal ion homeostasis, which is due to their high affinity for these metals, is mostly investigated [19,20]. For mammals, MTs bind zinc [21], but with excess copper or cadmium, zinc can be easily replaced by these metals [22]. Cells that contain excessive amounts of MTs are resistant to cadmium toxicity [23], while cell lines that cannot synthesize MTs are sensitive to cadmium [24]. Genetic studies using transgenic or knockout mouse models are further evidence of the role of MTs in protection against cadmium toxicity [25,26]. Based on structural models, it can be assumed that the MT molecule is composed of two binding domains, α and β, which are composed of cysteine clusters. Covalent binding of metal atoms involves sulfhydryl cysteine residues (Figure 1). The *N*-terminal part of the peptide is designated as β-domain and has three binding sites for divalent ions, and the C-terminal part (the α-domain) has the ability to bind four divalent metal ions.

Four mammalian MT isoforms (MT-1–MT-4) and 13 MT-like human proteins were identified [28]. The differences of constituent forms come mainly from post-translational modifications, small changes in primary structure, type of incorporated metal ion and speed of degradation. Despite the physical-chemical similarity of the forms, their roles and occurrence in tissues vary significantly [29]. MT-1 and MT-2 are present almost in all types of soft tissues [30–32], MT-3 is expressed mostly in brain tissue, but also in heart, kidneys and reproductive organs [33,34] and the MT-4 gene was detected in stratified squamous epithelial cells associated with oral epithelia, esophagus, upper stomach, tail, footpads and neonatal skin. [35]. In humans, the MT genes are located on chromosome 16 in a cluster and involve 16 identified genes, from which five are pseudogenes [36]. Although the MT-II, MT-III and MT-IV proteins are encoded by a single gene, the MT-I protein comprises many subtypes encoded by a set of 13 MT-I genes. The known active MT-I genes are MT-IA,-IB, -IE, -1F, -IG, -IH,-IM and -IX. The rest of the MT-I genes (MT-1C,-1D,-1I,-1J and 1L) are pseudogenes that are not expressed in humans [36].

## 3. Zinc as Signaling Compound and Antioxidant

Given that a number of MT functions are due to its close interaction with zinc ions, it is appropriate to mention zinc and metallothionein roles for easier comprehension separately. However, it should be noted that a separate description is too “textbook-styled”, while *in vivo* systems operate both systems simultaneously. The role of zinc has been extensively studied in recent years, and thus, the insight into this issue distinctly has changed during the last decade. Originally, the mere perception of zinc(II) as a structural component and an integral component of cytoskeletal structures significantly expanded, and these ions are considered as an important signaling component necessary for the physiological function of all cells [37–40]. These include influence on redox state, enzyme activity, gene transcription, energetic metabolism, cell cycle, cell migration and invasivity, apoptosis and proliferation [41–45]. Not surprisingly, disbalance in the levels of zinc, and thereby causing interference in these systems, has important consequences, including the development of cancers and other diseases [46]. The importance of zinc(II) might be illustrated by the consequences of the near-complete chelation of cellular zinc content. A potent zinc(II) chelator, TPEN (*N*,*N*,*N*′,*N*′-tetrakis[2-pyridylmethyl]ethylenediamine), for instance, induces cell death [47,48]. In contrast, cell death may be prevented by zinc(II) treatment and, thus, restore these unphysiological levels [46].

Due to the fact that zinc can’t freely pass through the membranes, the crucial role in the maintenance of intracellular zinc level is provided by zinc-transporting proteins, ZIPs (Zrt-Irt-like protein or Zinc Iron permease) and ZnTs (Zinc transporters) [49,50]. A concept of overall zinc signaling, either on the regulation of transcription or antioxidant acting, can be generalized to a scheme, where various stimuli (cell stress) lead to the increase in ROS/RNS and other oxidants, which subsequently release zinc(II) from a pool (usually MT), and this free zinc(II) fraction influences the target structures. In addition, a direct interaction between MT and apo-zinc binding peptides during the process of zinc transfer has been demonstrated in a cell-free system [51].

With regard to energetic metabolism, zinc(II) has inhibitory effects on the mitochondrial enzyme, aconitase, which catalyzes the conversion of citrate to isocitrate and, thus, enables the utilization of citrate in the Krebs cycle. This cascade is prostate-specific, and due to inhibition of this enzyme, prostate cells act as zinc-accumulating [52]. Also, the proapoptic effects of zinc are well-described. Zinc(II) facilitates the formation of BAX pores on the mitochondrial membrane and, thus, increases the BAX/Bcl-2 ratio. As a result, cytochrome c moves to the cytoplasm and triggers a caspase cascade, resulting in apoptosis [53,54]. Nevertheless, these aspects of zinc(II) are the subject of many comprehensive reviews [55–57], but the aim of this review lies in zinc’s relation to oxidative stress.

Although zinc(II) itself has no redox capacity, it is considered a potent and important antioxidant agent [45]. Cellular zinc decrease was associated with an increase in oxidants and oxidation parameters in several studies [58–61]. Its antioxidant properties are due to both the direct and indirect interference with target structures. These include induction of metallothionein expression and glutathione synthesis, regulation of oxidant production, association with cysteines (with concomitant release by other oxidants) and regulation of redox signaling.

Zinc(II) itself causes an increase in the major zinc-binding protein metallothionein. The induction of MT expression is induced through metal regulatory transcription factor 1 (MTF-1), a 753 amino acid transcription factor, which directly responds to increased levels of free zinc(II) [62]. Thus, MTF-1 binds the metal-responsive element of the *MT* gene and initiates MT transcription [63]. This autoregulatory loop maintains narrow optimal limits of intracellular zinc(II) and helps to reduce generated oxidative stress, as mentioned below.

In addition to zinc-metallothionein interactions widely discussed in this review, zinc(II) is an important regulator of glutathione (GSH) synthesis. The importance of zinc in the metabolism of glutathione underscores the finding that, as zinc deficiency is accompanied by oxidant increase, many studies reveal a deficiency of glutathione under such conditions [59,60]. Glutamate-cysteine ligase (GCL) was identified as a link in these conditions. GCL is a key regulatory enzyme in the synthesis of glutathione. It catalyzes a reaction of glutamate and cysteine to gamma-l-glutamyl-l-cysteine in glutathione and glutamate metabolism [64]. As shown on primary rat endothelial cells exposed to H_2_O_2_, zinc supplementation protects from peroxide-induced cell death via increasing the transcription of the GCLC and the concentrations of glutathione (GSH). Conversely, zinc depletion significantly decreased the expression of GCLC and the cellular GSH levels [65].

In addition, zinc deficiency was associated with indirect regulation of oxidant production. Although little is known, it was demonstrated that zinc(II) deficiency increased the level of NO and superoxide anion by the inhibition of the *N*-methyl-d-aspartate receptor (NMDAR) [45] As described on PC12 cells, NMDAR activation leads to an increase in cytoplasmic calcium levels and, thus, to increased ROS [66]. It is worth mentioning that zinc(II) is an integral part of up to 10% of all human proteins [67]. These proteins include zinc fingers, domains consisting mostly of cysteine and histidine able to interact with DNA specific bases [68]. The function of zinc fingers consists not only in DNA recognition and transcriptional activation, but also in RNA packaging, protein folding and apoptosis, whose regulation is important not only in development of tissues, but also in neoplastic transformation and proliferation [69,70]. For instance, zinc exchange between MT and zinc finger transcription factors [17,71,72] serves as a mechanism for the regulation of gene expression through activation or inhibition of DNA binding by estrogen receptors [73], SP1[71,74], TFIIIA [72,75], Gal4 [76] or tramtrack [77]. The above mentioned MTF-1-mediated MT expression is another *de facto* example of zinc finger transcriptional activity.

In addition to those “traditional” effects, Kröncke *et al.* attributed another function to zinc fingers; these structures act as redox-sensitive molecular switches. Similarly, as ROS or RNS cause the release of zinc(II) form metallothionein, it leads to a release of zinc(II) from these structures, causing not only a loss of zinc-finger function, but also an increase in cytoplasmic or nuclear free zinc(II) that may, in turn, stimulate and interfere with cellular signaling cascades [78].

## 4. Zinc and MT

**Figure 2:**
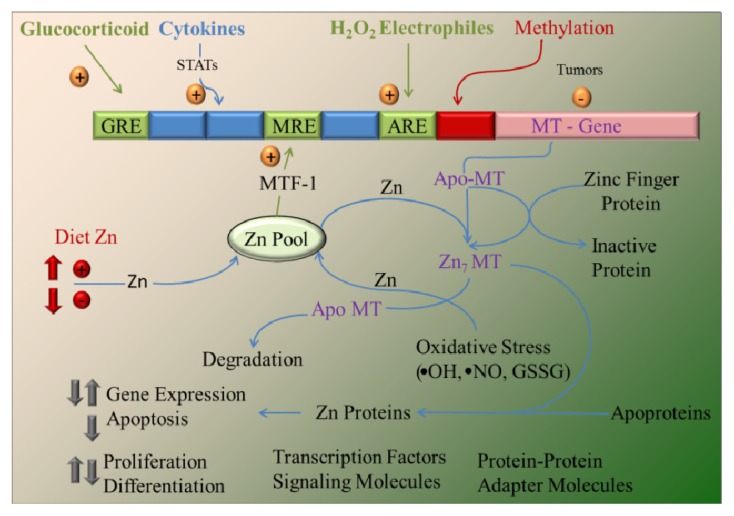
Overview of metallothionein (MT) gene regulation and function. The MT promoter has many response elements that upregulate transcription. These include the following: (1) metal response elements (MRE), which are activated by the metal-responsive transcription factor (MTF-1) after zinc occupancy, which is a function of the dietary zinc supply; (2) glucocorticoid response elements (GRE); (3) elements activated by STAT (signal transducers and activators of transcription) proteins through cytokine signaling; and 4) the antioxidant (or electrophile) response element (ARE), activated in response to redox status. Methylation may downregulate expression in some tumor cells. Cellular zinc pools are influenced by dietary zinc intake and zinc transporter activity and serve as the source of zinc bound to MT. Zinc bound to MT exhibits high thermodynamic stability. Apo-MT (thionein) and Zn_7_-MT (all coordination sites occupied) may serve to abstract or donate zinc, respectively, from/to zinc metalloproteins. Apo-MT is more rapidly degraded than Zn_7_-MT. The numerous zinc coordination sites of proteins (including transcription factors, signaling molecules and adapter molecules that use zinc fingers for protein-protein interaction) provide the opportunity for the cellular MT level to influence key processes, including gene regulation, cell proliferation and differentiation, signal transduction and apoptosis, as well as influence oxidative damage caused by oxidative stress and electrophiles. Adopted and modified according to [18].

The binding of zinc to MTs has proven to be a physiologically relevant. Several studies have produced strong evidence to support the idea that MTs function as zinc chaperones for the regulation of gene expression and activity of proteins, such as metalloproteins and metal-dependent transcription factors, as shown in Figure 2 and discussed in the following papers [79,80]. The binding of zinc to MTs is thermodynamically stable, which makes MTs an ideal zinc reservoir *in vivo*. The question is how MTs make zinc available for other molecules, including transcription factors and metalloproteins.

Maret *et al.*[76] showed that there is fast zinc exchange between MT isoforms 1 and 2 and also between MT2 and the zinc cluster in the Gal4 transcription factor [76]. Moreover, Jacob *et al.* found zinc transfer between MT and the apo-forms of zinc proteins *E. coli* alkaline phosphatase and bovine carboxypeptidase A [80]. Reduced glutathione (GSH) and glutathione disulfide (GSSG) are critical modulators of both the rate of zinc transfer and the ultimate number of zinc atoms transferred [79,81,82]. GSH inhibits zinc release in the absence of GSSG, indicating that MT is stabilized at relatively high cellular GSH concentrations. The presence of GSSG results in zinc release [81].

Under stress conditions, zinc release from MT occurs when the levels of nitric oxide or reactive oxygen species increase [83–86]. Treatment of lung fibroblasts with the NO donor, S-nitrosocysteine, resulted in an increase in intracellular labile zinc, as detected by a zinc-specific fluorophore, Zinquin, in wild-type, but not MT-null, fibroblasts [84]. Additional data obtained in sheep pulmonary artery endothelial cells suggested a role for the apo form of MT, thionein (T), as a Zn^2+^-binding protein in intact cells [84]. Collectively, these data showed that MT mediates NO-induced changes in intracellular Zn(II). Furthermore, in another study, Pearce *et al.* have shown that, in cultured pulmonary artery endothelial cells, a MT-green fluorescent fusion protein (FRET-MT) undergoes conformational changes in the presence of NO [87]. These conformational changes are consistent with the release of metals from the thiolate clusters of MT [88–90].

## 5. The Role of MT in Cancer and Apoptosis

**Figure 3:**
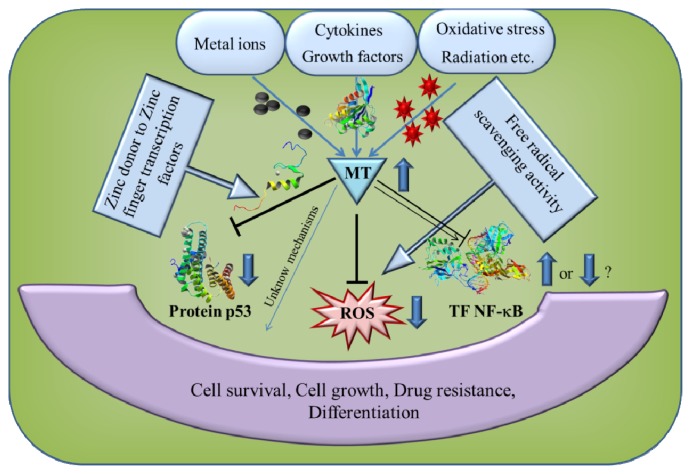
Schematic presentation of the stimuli that induce MT and the downstream effects of MT overexpression. MT can be activated by a variety of stimuli, including metal ions, cytokines, growth factors, oxidative stress and radiation. Downstream effects of MT overexpression are modulation of transcription of both tumor suppressor protein p53 and nuclear transcription factor NF-κB. Another downstream effect of MT overexpression is free radical scavenging activity. All these downstream MT effects influence cell survival, cell growth, drug resistance and differentiation. Adopted and modified according to [107].

MT can be activated by a variety of stimuli, including metal ions, cytokines and growth factors [91–93], as shown in Figure 3. In a number of experiments, the synthesis of MT was shown to be increased by several-fold during oxidative stress [94,95] to protect the cells against cytotoxicity [96,97], radiation and DNA damage [98–100]. The stimuli that induce MT and the downstream effects of MT overexpression are summarized in Figure 3. A lot of studies have shown an increased expression of MT in various human tumors of the breast, colon, kidney, liver, lung, nasopharynx, ovary, prostate, salivary gland, testes, thyroid and urinary bladder [91]. MT expression in tumor tissues is mainly correlated with the proliferative capacity of tumor cells [101]. However, there are few exceptional cases, like downregulation of MT-I and -II in hepatocellular carcinoma [102] and also a reduced level of intracellular zinc, resulting in the increase of granulocytes, but a decreased number of lymphocytes [103]. Hence, the expression of MT is not universal to all human tumors, but may depend on the differentiation status and proliferative index of tumors, along with other tissue factors and gene mutations. Downregulation of MT synthesis in hepatic tumors may be related to hypermethylation of the MT-promoter or mutation of other genes, such as the p53 tumor suppressor gene. Mao *et al.*[104] identified a member of the MT family, termed *MT1M*, which is expressed in various normal tissues, with the highest level in the liver. However, *MT1M* expression markedly decreased in human hepatocellular carcinoma specimens. A methylation profiling analysis indicated that the MT1M promoter is methylated in the majority of hepatocellular carcinoma tumors examined. Moreover, restored expression of *MT1M* in the hepatocellular carcinoma cell line, Hep3B, which lacks endogenous *MT1M* expression, suppressed cell growth *in vitro* and *in vivo* and augmented apoptosis induced by tumor necrosis factor [104]. Similarly, Yan *et al.*[105] observed downregulation of *MT1F* by loss of heterozygosity in colon cancer tissue. Furthermore, exogenous *MT1F* expression increased colon cancer cell line (RKO) apoptosis and inhibited RKO cell migration, invasion and adhesion, as well as *in vivo* tumorigenicity. From this study, it could be concluded that *MT1F* is a putative tumor suppressor gene in colon carcinogenesis [105]. In another study, Faller *et al.*[106] observed DNA methylation in *MT1E* in malignant melanoma, which suggests that *MT1E* is also a potential tumor suppressor gene.

MTs can also help cancer cells to survive by inhibition of apoptosis [108,109]. Apoptosis, or programmed cell death, is a mechanism for the elimination of unnecessary or damaged cells [110]. This process involves the activation of cysteine proteases caspases and, subsequently, of nuclear endonucleases. These events later lead to nuclear DNA damage and to ceasing of all biosynthetic processes in the cell [111]. During apoptosis, in contrast to necrosis, the rupture and spillage of cell content, which would cause inflammation, does not occur, but cells undergo condensation, DNA fragmentation and disintegration of the cell into small parts that can be easily phagocytosed [112,113]. Deregulation of apoptosis is essential for pathogenic mechanisms in many diseases, such as neurodegenerative disorders, autoimmune disease and cancers [113–115].

MT plays two important roles in the regulation of apoptosis. The first role of MT is regulation of intracellular zinc concentration, and the second role is interaction of MT with some proteins involved in apoptosis. MT protects against apoptosis by distributing cellular Zn. Zinc is an intracellular mediator of apoptosis, which can interfere with the action of Ca^2+^. Zinc addition prevents DNA fragmentation and inhibits many proteins connected to apoptosis, such as caspases and calcium-magnesium–dependent proteases [116]. Increased apoptosis *in vivo* may occur as a direct or indirect consequence of a decrease in intracellular Zn concentration [117,118]. Therefore, cellular Zn is described as an inhibitor of apoptosis, while its depletion induces death in many cell lines [119]. This Zn depletion activates caspases-3, -8 and -9, responsible for the proteolysis of several target proteins, like poly (ADP-ribose) polymerase or transcription factors [120]. Moreover, zinc is involved in structural stabilization and activation of the p53 that appears to be an important component of the apoptotic process by inducing a transcription of the p53 gene, with increased expression of p53 mRNA and protein [121].

The tumor suppressor p53 protein is a metal-binding transcription factor, which binds DNA through a structurally complex domain stabilized by a zinc atom [122,123]. The nuclear accumulation of MT may be important for supplying zinc or other metals to target molecules, including enzymes, zinc-finger transcription factors and tumor suppressor gene products, such as p53 [31,73,122–125]. Meplan *et al.*[123] demonstrated that zinc incorporation is required for the stabilization of wild-type recombinant p53 in a form capable of binding specifically to DNA. They also showed that human recombinant thionein, the metal-free form of MT, reported to remove zinc from zinc finger transcription factors Sp1, thereby abrogating their transcriptional activity [74,75], inhibited binding of p53 to a specific consensus sequence *in vitro*. Supplementation of thionein with equimolar amounts of zinc prior to incubation with p53 abrogated this effect. Further, recombinant MT, a metal-chelator protein, was found to modulate p53 conformation *in vitro*. In cultured cells, overexpression of MT by transfection could modulate p53 transcriptional activity [123]. Analysis of human cancer patients also showed interesting correlations between p53 and MT gene expressions. In pancreatic serous cystadenomas, the increased expressions of MTs and p53 were observed in the less-differentiated tumors [126]. Similarly, in oral squamous cell carcinoma, frequent localization of MTs in nuclei was associated with the increased expression of the p53 gene [127].

One of the most important MT interactions with proteins involved in apoptosis is the regulation of NF-κB activity. Nuclear factor-κB (NF-κB) is a transcription factor that is involved in the regulation of cell death. Overexpression of NF-κB renders cancer cells resistant to chemotherapeutic agents [128,129], and it has been suggested that the antiapoptotic proteins IAP, IEX-1L and the Bcl-2 family are regulated by NF-κB transcription [130–132]. MT-1 and MT-2 regulate the level, activity and cellular location of the transcription factor, NF-κB [133–136]. In fact, MT interacts with the p50 subunit of NF-κB to increase the transactivation of NF-κB [135]. In another study, MT overexpression was found to upregulate NF-κB DNA binding [137]. Those interactions are important for the growth of some tumors, e.g., activation of NF-κB may mediate the antiapoptotic effect of MT.

## 6. Antioxidant Function of MT

**Figure 4:**
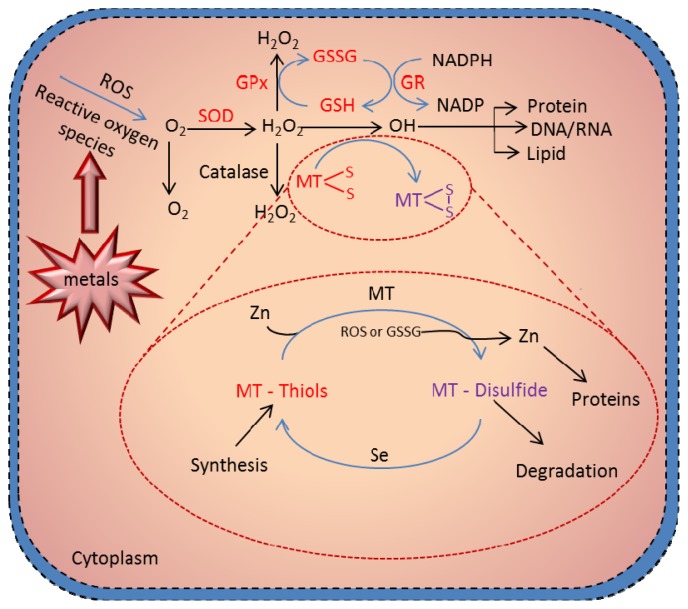
Metallothionein scavenging of reactive oxygen species. Presence of redox metals, such as Cu and Fe, in a cell can produce reactive oxygen species (ROS), leading to damaging of DNA and cell structures. The cell protects itself using various molecules as scavengers of the radicals. One of the most crucial cell pathways to scavenge the radicals is the glutathione redox complex. However, free –SH moieties of MT can be also involved in the scavenging of ROS in the MT redox cycle. Under physiologic conditions, zinc bound to MT is released through oxidation of the thiolate cluster when the environment becomes oxidized. Formation of MT-disulfide would be subjected to degradation; however, when the oxidized environment became reduced—through, for example, an increase in the glutathione (GSH)/glutathione disulfide (GSSG) ratio—MT disulfide is reduced to MT-thiol. This reduction process is greatly enhanced in the presence of selenium catalyst. In the presence of zinc, MT is quickly reconstituted. This process constitutes the MT redox cycle, which plays a crucial role in the biologic function of MT. Adopted and modified according to [141] and [93].

The most critical advance in MTs research is the demonstration of the redox regulation of Zn-S interaction and the coupling of zinc and redox metabolism [88]. The cluster structure of Zn-MT provides a chemical basis by which the cysteine ligand can induce oxidoreductive properties [89]—what constitutes a MT redox cycle (Figure 4). The hypothesis that MT functions as an antioxidant against reactive oxygen and nitrogen species has received extensive experimental support from many *in vitro* studies. Studies using a cell-free system have demonstrated the ability of MT as a free radical scavenger [138–140]. Metallothionein has been shown to scavenge hydroxyl radicals *in vitro*, because of its cysteinyl thiolate groups [94] Thornalley and Vasak [138] showed that the rabbit liver metallothionein-1, which contains zinc and/or cadmium ions, appeared to scavenge free hydroxyl (•OH) and superoxide (O^2−^•) radicals produced by the xanthine/xanthine oxidase reaction. All 20 cysteine sulfur atoms are involved in the radical quenching process, and the rate constant for the reaction of hydroxyl radical with MT is about 340-fold higher than that with GSH [138].

Studies using cultured cells and intact animal models have provided further evidence supporting the antioxidant function of MT [95,100,142–147]. Quesada *et al.*[148] examined the reaction of the sulfhydryl groups in metallothionein with hydrogen peroxide in human promyelocytic leukemia cells (HL-60). Zinc-metallothionein (Zn-MT) was induced by 24-h treatment of HL-60 cells with ZnCl^2^.The ratio of H_2_O_2_ concentrations needed to reduce HL-60 cell survival by 50% in Zn-MT-induced cells compared to normal cells was 1.65 to 1. So, the Zn-MT-induced cells were more resistant to oxidative stress caused by hydrogen peroxide than normal cells. In the other paper Chubatsu *et al.*[149] investigated the role of MT in protection against oxidative damage to DNA on V79 Chinese hamster cells. An increase in MT content of V79 Chinese hamster cells was induced by zinc without concomitant increase in the GSH level. These induced cells were more resistant to the production of DNA-strand scission by H_2_O_2_ than the parental cells. Conversely, cells rendered partially deprived of MT, by transfection with a plasmid vector in which the MT-I cDNA is antisense oriented in relation to a simian virus 40 promoter, became more susceptible to the DNA-damaging action of H_2_O_2_[149]. In another study, Schwarz *et al.*[142] examined the sensitivity of NIH 3T3 cells transfected with a plasmid containing mouse metallothionein-I gene (NIH3T3/MT) to the membrane permeant oxidant, tert-butyl hydroperoxide. NIH3T3/MT cells had a four-fold increase in intracellular metallothionein, as compared to cells transfected with a plasmid containing an inverted gene (NIH3T3/TM). NIH3T3/MT cells were six-times more resistant than NIH3T3/TM cells to the cytotoxic effects of tert-butyl hydroperoxide. Furthermore, homogenates of NIH3T3/MT cells were more capable of scavenging *in vitro*-generated phenoxyl radicals, as quantified by electron spin resonance detection.

MT is primarily localized in the cytoplasm [150]. The highest cytoplasmic concentration of MT was found in the late G1 and G1/S cell cycle phase [151]. Depending on the cell cycle phase, cell differentiation or in the case of toxicity, MT-1 and MT-2 are rapidly translocated to the nucleus, as seen in oxidative stress and during the early S-phase [151–153]. It has been reported that hydrogen peroxide induces the nuclear localization of MT in culturing BALB 3T3 cells, depending on the cell cycle [154,155]. Karyophilic MT induced by H_2_O_2_ treatment was suggested to play the role of nuclear antioxidant [156]. Moreover, Ogra *et al.*[157] reported that nitric oxide enhances the nuclear localization of MT in digitonin-permeabilized semi-intact HeLa cells. The results suggest that MT can scavenge NO using the sulfhydryl groups of cysteines in its molecule to form nitrosothiol, thereby reducing nuclear and cytoplasmic damage by NO. In the next study, Du *et al.*[143] examined the role of reactive oxygen metabolites and the protective effect of zinc-induced MT synthesis on gentamicin nephrotoxicity in rats, both *in vivo* and *in vitro*. In the *in vivo* study, MT content of the renal cortex of the zinc preinjected rats was significantly increased, and proximal tubular necrosis and acute renal failure caused by injection of gentamicin were ameliorated. In suspended proximal tubules (PT), Na^+^-K^+^-ATPase activity and DNA synthesis were suppressed by the addition of gentamicin, but in zinc-pretreated rats’ PT, these were not suppressed by the addition of gentamicin. In addition, malondialdehyde and hydroxyl radical production in Zn-preinjected rats’ PT were significantly lower than those in the normal and saline-preinjected rats’ PT. In another study, Sato *et al.*[158] determined dose-dependent changes in the concentration of metallothionein-I (MT-I) in rat tissues following subcutaneous administration of paraquat (PQ), a superoxide radical-generating agent. Twenty four hours after injection, MT-I concentrations in the lung increased linearly with PQ dose. Concentrations in the liver increased with dose, until a plateau was reached at a dose of 30 mg/kg body wt. In the kidneys, MT-I concentrations did not increase, even at high doses of PQ. Zn was the principal metal bound to MT in the liver. The same authors [159] studied the roles of cytokines tumor necrosis factor (TNF) and interleukin 6 (IL-6) in MT synthesis induced by the superoxide generator, paraquat (PQ). They came to the conclusion that MT synthesis induced by oxidative stress may be, at least partly, mediated through cytokines, because pretreatment of rat with dexamethasone, an inhibitor of cytokine production, prevented MT synthesis induced by paraquat.

In addition, MT was reported to be induced by radiation. Shiraishi *et al.*[160] measured hepatic and renal MT contents in rats following whole-body X-irradiation. When compared with control rats, the hepatic MT-Zn content increased five-fold, and MT protein content increased 15-fold by 18 h following irradiation. Similar results were reported from Koropatnick *et al.*[161]. They observed that whole-body X irradiation of mice induces MT-1 mRNA transcription and protein expression and accumulation in liver, but not in kidney or spleen. Similarly, Shibuya *et al.*[162] examined the accumulation of MT in the Meth-A tumor (mouse fibrosarcoma cells) transplanted into mice exposed to whole-body X irradiation. The MT content in the tumor cells was increased by X irradiation in a dose-dependent manner. Matsubara *et al.*[163,164] found a striking radioresistance in mice, which were subjected to various pretreatments to induce MT synthesis in the liver prior to irradiation. The normal level of MT in mouse liver is 20 μg/g tissue. This level increased up to 70 μg/g tissue following irradiation at 6.3 Gy. Among irradiated mice, MT levels in the liver increased approximately 200%–800% after cadmium, manganese or zinc injection, compared to levels of irradiated mice without pretreatment. The observed results suggest that the body’s protective mechanism against radiation strongly correlates with the biosynthesis of MT or MT itself acting as a scavenger of radiation-induced peroxides. On the other hand, MT-transgenic mice, which carried 56 copies of the MT-I transgene and had higher tissue MT concentrations, were not protected against the toxic effects produced by gamma-radiation [165].

The most convincing evidence for the antioxidant action of MT was generated from genetically manipulated mouse model studies. Using MT-overexpressing transgenic or MT-null mice, many studies have shown MT protection against oxidative injuries induced by a diversity of oxidative conditions, including doxorubicin cardiotoxicity, ischemia/reperfusion, diabetes and alcohol administration [166–170].

Sun *et al.*[166] investigated the effect of overexpression of MT on doxorubicin chronic cardiotoxicity in mice, since MT is a potent antioxidant and oxidative stress is critically involved in doxorubicin-induced heart injury. As compared with nontransgenic controls, doxorubicin-induced cardiac hypertrophy was significantly inhibited in the transgenic mice. Light microscopic examination revealed that doxorubicin-induced myocardial morphological changes were markedly suppressed or almost eliminated in the transgenic mice. In the next study using also a cardiac-specific MT-overexpressing transgenic mouse model, Kang *et al.*[167] demonstrated that MT suppresses ischemia/reperfusion-induced myocardial apoptosis through, at least in part, the inhibition of the cytochrome c-mediated caspase-3 activation pathway. The antiapoptotic effect of MT likely results from the suppression of oxidative stress and correlates with the inhibition of myocardial infarction. Another study with the same transgenic mice model was performed to test whether inhibition of nitrosative damage is involved in MT prevention of diabetic cardiomyopathy [168]. Cardiac-specific MT-overexpressing transgenic mice and wild-type controls were treated with streptozotocin by a single intraperitoneal injection, and both developed diabetes. However, the development of diabetic cardiomyopathy, revealed by histopathological and ultrastructural examination, serum creatine phosphokinase and cardiac hemodynamic analysis, was significantly observed only in the wild-type, but not in MT-overexpressing transgenic, diabetic mice. Formations of superoxide and 3-nitrotyrosine, a marker for peroxynitrite-induced protein damage, were detected only in the heart of wild-type diabetic mice [168]. These results thus suggest that MT prevention of diabetic cardiomyopathy is mediated, at least in part, by suppression of superoxide generation and associated nitrosative damage. In addition, Wang *et al.*[169] showed that MT-null mice are more prone to develop cardiac hypertrophy and fibrosis after feeding an alcohol-containing liquid diet for two months than the wild-type control mice.

In another study, Zhou *et al.*[171] evaluated MT-mediated cardioprotection from angiotensin II-induced pathologic remodeling in both the nondiabetic and diabetic heart. The acute and chronic cardiac effects of angiotensin II were examined in MT-overexpressing transgenic and wild-type mice, and the signaling pathways of angiotensin II-induced cardiac cell death were examined in neonatal mouse cardiomyocytes. Acute angiotensin II administration to wild-type mice or neonatal cardiomyocytes increased cardiac apoptosis, nitrosative damage and membrane translocation of the nicotinamide adenine dinucleotide phosphate oxidase (NOX) isoform p47 (phox). These effects were abrogated in MT-overexpressing transgenic mice and MT-overexpressing transgenic cardiomyocytes. In addition, prolonged administration of angiotensin II also induced apoptosis and nitrosative damage in both diabetic and nondiabetic wild-type hearts, but not in diabetic and nondiabetic MT-overexpressing transgenic hearts.

An interesting result came from a study with a MT gene family knockout in *Drosophila melanogaster*. Egli *et al.*[172] reported the generation of viable flies with targeted disruption of all four MT genes (MtnA, MtnB, MtnC and MtnD). These flies were highly sensitive to copper, cadmium and, to a lesser extent, zinc load during development. MT expression was particularly important for male viability. While copper load during development affected males and females equally, adult males lacking MTs displayed a severely reduced life span, possibly due to copper-mediated oxidative stress. Another finding of the study was that MTs are expressed in a tissue-specific manner, notably at sites of metal accumulation. Binding of copper to MTs leads to an orange luminescence of copper accumulated in copper cells of the midgut. Once heavy metal is bound in such a manner, it no longer triggers the activation of MT genes, thus generating a negative feedback on MT gene expression [172].

## 7. Conclusions

Oxidative or nitrosative stress can cause a release of zinc from proteins containing zinc-fingers and cluster motifs and its re-distribution, thereby altering the functions of those proteins from which it is released and/or to which it binds. Based on the above mentioned facts, MTs belong to the important maintainers of the zinc pool and, also, can be associated with scavenging of free radicals. It is clear that the potential of MT as a scavenger of reactive species is not fully understood, but the published data show that this protein could be selected as a target for some treatment strategies, mainly in the case of tumor diseases. In addition to this, MT overexpression could be used as a predictive marker of worse prognosis and a sign of a higher grade in selected tumors [173].

## Figures and Tables

**Table 1:** Summary of reactive oxygen and nitrogen species [10,11].

Reactive Oxygen Species				Reactive Nitrogen Species			
	
Free Radicals		Other Substances		Free Radicals		Other Substances	
Superoxide anion radical	O_2_•−	Hydrogen peroxide	H_2_O_2_	Nitric oxide radical	NO•	Peroxynitrite	ONOO^−^
Hydroxyl radical	HO•	Hypochlorous acid	HOCl	Nitric dioxide radical	NO_2_•	Nitrites	NO_2_^−^
Alkoxyl radical	RO•	Ozone	O_3_			Nitrates	NO_3_^−^
Peroxyl radical	ROO•	Singlet oxygen	^1^O_2_			Nitrosyl	NO^+^
